# Analysis of the galactomannan binding ability of β-mannosidases, BtMan2A and CmMan5A, regarding their activity and synergism with a β-mannanase

**DOI:** 10.1016/j.csbj.2022.06.038

**Published:** 2022-06-17

**Authors:** Samkelo Malgas, Mariska Thoresen, Vuyani Moses, Earl Prinsloo, J. Susan van Dyk, Brett I. Pletschke

**Affiliations:** a Enzyme Science Programme (ESP), Department of Biochemistry and Microbiology, Rhodes University, Makhanda, Eastern Cape 6140, South Africa; b Department of Biochemistry, Genetics and Microbiology, University of Pretoria, Hatfield, Gauteng 0028, South Africa; c Research Unit in Bioinformatics (RUBi), Department of Biochemistry and Microbiology, Rhodes University, Makhanda, Eastern Cape 6140, South Africa; d Biotechnology Innovation Centre, Rhodes University, Makhanda, Eastern Cape 6140, South Africa; e Forest Products Biotechnology, University of British Columbia, 2424 Main Mall, Vancouver, British Columbia V6T1Z4, Canada

**Keywords:** β-mannosidase, Galactomannan, Non-catalytic binding

## Abstract

Both β-mannanases and β-mannosidases are required for mannan-backbone degradation into mannose. In this study, two β-mannosidases of glycoside hydrolase (GH) families 2 (BtMan2A) and 5 (CmMan5A) were evaluated for their substrate specificities and galactomannan binding ability. BtMan2A preferred short *manno*-oligomers, while CmMan5A preferred longer ones; DP >2, and galactomannans. BtMan2A displayed irreversible galactomannan binding, which was pH-dependent, with higher binding observed at low pH, while CmMan5A had limited binding. Docking and molecular dynamics (MD) simulations showed that BtMan2A galactomannan binding was stronger under acidic conditions (-8.4 kcal/mol) than in a neutral environment (-7.6 kcal/mol), and the galactomannan ligand was more unstable under neutral conditions than acidic conditions. Qualitative surface plasmon resonance (SPR) experimentally confirmed the reduced binding capacity of BtMan2A at pH 7. Finally, synergistic β-mannanase to β-mannosidase (BtMan2A or CmMan5A) ratios required for maximal galactomannan hydrolysis were determined. All CcManA to CmMan5A combinations were synergistic (≈1.2-fold), while combinations of CcManA with BtMan2A (≈1.0-fold) yielded no hydrolysis improvement. In conclusion, the low specific activity of BtMan2A towards long and galactose-containing oligomers and its non-catalytic galactomannan binding ability led to no synergy with the mannanase, making GH2 mannosidases ineffective for use in cocktails for mannan degradation.

## Highlights

• BtMan2A preferred short *manno*-oligomers, while CmMan5A preferred longer ones; DP >2.
• BtMan2A displayed stronger irreversible binding to galactomannan than CmMan5A.
• BtMan2A binding to galactomannan did not affect its activity, while CmMan5A lost activity.
• BtMan2A binding was pH-dependent, with increased binding ability at lower pH.
• CmMan5A synergised with CcManA, while BtMan2A did not – even though the enzyme was active.
• High loadings of BtMan2A abolished CcManA activity; at protein ratios ≥ 5:1.

## Graphical abstract

**Figure:**
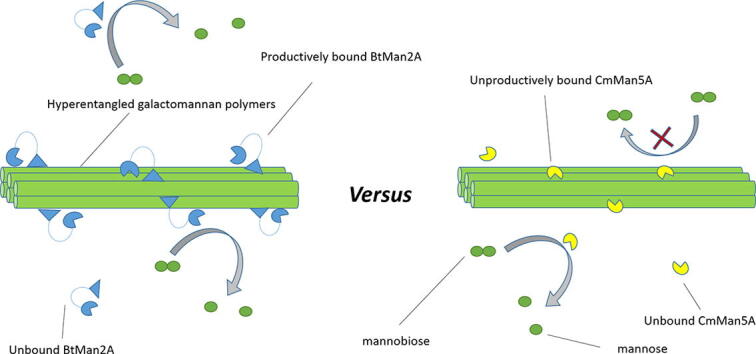

## 1 Introduction

Mannans are one of the major constituents of hemicellulosic polysaccharides and are found in various plant tissues, including wood, tubers, beans and endosperm seeds [1]. Their primary physiological roles include: acting as structural components of plant cell walls by forming a complex network with cellulose, as storage polysaccharides in endosperm seeds, and vacuoles of vegetative tissues [2], [3], [4]. The mannan backbone is composed of various sugars, such as D-mannose and/or D-glucose, and can be either linear or branched with α-1,6 linked D-galactosyl units [1], [5]. The four major classes of mannans are linear mannan, glucomannan, galactomannan, and galactoglucomannan [1], [4]. Galactomannan is the preferred substrate for carbohydrate research since galactoglucomannan is not readily available commercially [6]. The main sources of galactomannan are locust bean, carob tree and guar tree [7].

The efficient depolymerisation of mannan is achieved by the synergistic action of a consortium of glycoside hydrolases (GH), that are grouped into respective GH families according to their amino acid sequences, structural properties and mechanistic behaviours [5], [7]. The three key enzymes involved in mannan backbone hydrolysis are *endo*-β-1,4-D-mannanase (β-mannanase, EC 3.2.1.78), *exo*-1,4-mannobiohydrolase (EC 3.2.1.100) and 1,4-β-D-mannosidase (β-mannosidase, EC3.2.1.25) [8], [9], [10]. β-Mannanases are responsible for cleaving the β-1,4-linkages in the main chain to liberate mannooligosaccharides and create new chain ends [7], while mannobiohydrolases remove mannobiose residues from the non-reducing ends of mannan chains [9]. The mannooligosaccharides are further hydrolysed to mannose by β-mannosidase, an *exo*-acting enzyme that releases mannose from the non-reducing ends of mannan and mannooligosaccharides [5]. These three enzyme classes are essential for the complete hydrolysis of the mannan backbone and are found in GH families 1, 2, 5, 26, 45, 113, 134 and 164 (clan GH-A) (www.cazy.org).

GH family 2 mannosidases; including mouse β-mannosidase, *Bacteriodes thetaiotaomicron* BtMan2A, *Trichoderma harzianum* ThMan2A and *Xanthomonas axonopodis* pv. *citri* XacMan2A; have a conserved domain organization consisting of five domains, with the catalytic domain positioned in the centre [11], [12], [13], [14]. On the other hand, the GH5 mannosidases, such as *Cellulomonas mixtus* CmMa5A and *Absidia corymbifera* AcMan5, have a simpler domain organization consisting of an active site region only [15], [16]. The two putative galactomannan binding pockets that were identified on ThMan2A by Nascimento et al. [12] were also observed on BtMan2A when the two mannosidases were super-positioned onto each other. The first galactomannan binding pocket is found between the interfaces of Domains 1, 2 and 3, while the second pocket is located at the interface between Domains 3 and 4. Based on sequence alignment data, four of the key residues in ThMan2A (D430, D434, D437 and H478) are conserved as D358, D362, D365 and H407, respectively, in *Caldicellulosiruptor bescii* CbMan2A and as D418, D422, D425 and H466, in XacMan2A (Supplementary Fig. A). These residues are involved in galactomannan binding in the first pocket, and the last three residues appear to also be conserved as D384, D387 and H428 in BtMan2A, while the first residue, D430 in ThMan2A, is replaced with N380 in BtMan2A [12], [17].

The rate at which enzymatic hydrolysis occurs is governed by the capacity of an enzyme to adsorb to the substrate, allowing the enzyme's catalytic site to be near the substrate's cleavage site [18], [19]. Traditionally, the phenomenon of protein adsorption has been investigated by Langmuir-type isotherms, whereby measurement of the overall protein adsorption is conducted under the assumption that the lignocellulosic substrate is a uniform adsorbent surface. Investigating the relationship between adsorption and mannan hydrolysis could potentially provide novel insights regarding the substrate specificities of the mannanolytic enzymes and their mechanisms of action. This information would be fundamental in our understanding of mannanolytic enzyme-substrate recognition and attack patterns, as well as in our understanding of their synergism during mannan hydrolysis.

Therefore, we comparatively evaluated the influence of reversible and irreversible adsorption of mannosidases, BtMan2A and CmMan5A, during their interaction with locust bean gum (LBG) galactomannan. The study showed that BtMan2A could bind to galactomannan via a non-catalytic binding site which does not affect its activity. In contrast, CmMan5A bound randomly with an extreme loss in activity as a result. There is also limited knowledge available on the sequence homology classification of mannosidases (GH2 or GH5) to the structural, functional and synergistic abilities of these enzymes with mannanases during galactomannan degradation. We also showed that synergism occurred between CmMan5A and a mannanase, CcManA; however, BtMan2A did not synergise with the mannanase. Synergism between the mannanase and mannosidases appeared to be governed by both the substrate specificity of the mannosidases with regards to the mannanase-produced *manno*-oligosaccharides and their galactomannan binding ability.

## 2 Methods

### 2.1 Materials

The mannosidases (BtMan2A and CmMan5A) were purchased from Prozomix (United Kingdom), while the mannose determination kit (K-MANGL), mannose (M1), mannobiose (M2), mannotriose (M3), mannotetraose (M4), mannopentaose (M5), mannohexaose (M6), guar gum (GG) and galactosyl-substituted mannooligosaccharides (α-6^1^-galactosyl-mannotriose and α-6^4^, 6^3^-di-galactosyl-mannopentaose) were purchased from Megazyme (Wicklow, Ireland). All other chemicals were reagent grade and purchased from Sigma-Aldrich (St Louis, United States of America). The mannanase (CcManA, GH5) from *C. cellulovorans* was expressed and purified in-house as described previously according to Beukes et al [20].

### 2.2 Protein determination

The protein concentrations of the enzymes were estimated using the Bradford assay [21]. Bovine serum albumin (BSA) was used as a suitable protein standard.

### 2.3 Substrate specificity determination

The activities of the mannosidases (BtMan2A and CmMan5A) were measured using *p*-nitrophenyl-β-D-mannopyranoside (*p*NPM) as a substrate. An appropriately diluted enzyme solution (50 µl) was mixed with 450 µl of 2.25 mM *p*NPM in 50 mM sodium citrate buffer (pH 5.0). The reaction was conducted at 37 °C for 15 min and was terminated by the addition of 500 µl of 2 M sodium carbonate. The released *p*-nitrophenyl product was measured at 405 nm, and the activity was determined from a *p*-nitrophenyl standard curve. The mannosidase activities were also determined on the polymeric galactomannan substrates at 0.5% (w/v) (GG and locust bean gum (LBG)) and 2 mM galactosyl-substituted mannooligosaccharides (α-6^1^-galactosyl-mannotriose and α-6^4^, 6^3^-di-galactosyl-mannopentaose). The reactions were conducted at 37 °C using 50 mM citrate buffer for 60 min. The reaction mixtures were then centrifuged at 16,060× *g* (Heraeus Biofuge pica micro-centrifuge) for 3 min. Mannose released by the enzymes on the galactose-containing oligomers and polymers was monitored using a mannose determination kit. The assay was monitored using a 96 well plate at 340 nm for 20 min with readings taken at one minute intervals (at 25 °C).

The activity of the *C. cellulovorans* mannanase, CcManA, was determined as described by Malgas et al. [6] using appropriate galactomannan substrates. All enzyme hydrolysis assays were run in triplicate. One unit of enzyme activity was defined as the amount of enzyme required to liberate 1 µmol of product per minute, under the assay conditions specified. The reducing sugar released was monitored with the DNS (dinitrosalicylic acid) method described by Miller [22]. A single standard curve for the DNS assay was prepared using mannose as a suitable standard.

### 2.4 Mannosidase adsorption isotherms

#### 2.4.1 Bradford's binding assays

Adsorption of BtMan2A and CmMan5A on galactomannan (LBG) was monitored by the Bradford method. Eppendorf tubes with 0.5% (w/v) galactomannan, suspended in 50 mM sodium citrate buffer (pH 5.0) were used. Hydrolysis was carried out at 25 °C on a rotor at 25 rpm. The reaction was started by adding 100 µl of mannosidase to 300 µl of galactomannan suspension. At selected time points, the hydrolysate was centrifuged (16,060× *g*) for 3 min using a benchtop centrifuge (Heraeus Biofuge pica micro-centrifuge), washed once with one part of 50 mM sodium citrate buffer (pH 5.0), pre-incubated at 25 °C. The protein concentration in the supernatant was determined as described above. The amount of total adsorbed mannosidase, the sum of both the reversibly and irreversibly adsorbed protein, was calculated by subtracting the amount of free enzyme in the first supernatant from the original amount of the added enzyme. The enzyme that remained on the substrate after the washing step (according to the above-mentioned protocol) was regarded as the irreversibly adsorbed enzyme. The amount of irreversibly adsorbed enzyme was then calculated by subtracting the total amount of free enzyme in all the supernatants (reaction supernatant and supernatant from the wash) from the original amount of enzyme added. Substrate (containing only the substrate without the enzyme) and enzyme controls (containing only the enzyme without the substrate) were also run and subjected to the Bradford method to determine substrate background detection and unbound protein, respectively.

The effect of acidic and neutral to alkaline pH on the binding of the mannosidase, BtMan2A, with galactomannan was studied by dissolving the galactomannan in 50 mM sodium citrate-phosphate buffer (pH 3.0, 5.0 and pH 7.0) and 50 mM Tris-HCl buffer at pH 9.0, respectively. Adsorption studies were conducted as described previously. Each set of experiments was carried out in triplicate. BSA was used as a suitable nonspecific binding reference protein in all binding assays.

#### 2.4.2 In silico analysis of BtMan2A and galactomannan interaction at varying pH

AutoDock Vina-Carb [23] was used to perform docking of a galactomannan substrate to the putative binding site on BtMan2A, determined in a previous study [12]. Molecular Dynamics (MD) simulations were then used to assess the stability of the interaction between BtMan2A and galactomannan at varying pH.

The protocol used for molecular docking involved obtaining the correct BtMan2A crystal structure (PDB id: 2EJ8) from the protein databank (PDB) (https://www.rcsb.org/). Once the structure was obtained, it was inspected for structural defects and then cleaned by removing crystallographic waters and other co-crystalized ligands. The structure was protonated in two different aqueous environments (pH 5.0 and pH 7.0) using the Proteins Plus server (https://proteins.plus/). Then a suitable galactomannan representative structure (PubChem CID: 439336) was obtained from PubChem [24] and docking was performed on the putative binding site using Vina-Carb. The Box dimensions used for the docking were ×  = 78 Å, y = 68 Å and z = 78 Å. The grid was then centred at ×  = 16.799 Å, y = 46.424 Å and z = 11.32 Å. An overall exhaustiveness of 100 was used for docking, and the binding energies produced were then used to identify the optimal binding poses of the galactomannan polysaccharide in the galactomannan binding site of BtMan2A.

Molecular Dynamics simulations were performed for both BtMan2A structures subjected to aqueous environments at pH 5.0 and pH 7.0 using GROMACS [25]. The force field used for the simulation was CHARMM 37. This force field was selected due to its inclusion of parameters for both proteins and carbohydrates, making it ideal for this analysis. The systems were simulated in a cubic box with a minimum distance of 1.0 Å from the edge of the protein. Energy minimization was then performed for 50,000 steps using a conjugate gradient. The temperature in the system was equilibrated at 310.15 K using a Berendsen thermostat [26], while the pressure was equilibrated at 1 bar using the Parrinello − Rahman barostat [27]. Both equilibration steps were performed for 100 ps. A simulation was performed of the galactomannan polysaccharide in the galactomannan binding site of BtMan2A at pH 5.0 and pH 7.0 for 100 ns using 240 cores (CPU: Intel® Xeon®). This experiment was carried out at the Centre for High-Performance Computing (Cape Town, South Africa). The Root Mean Squared Deviation (RMSD) of the protein, RMSD of the galactomannan polysaccharide, and Root Mean Squared Fluctuation (RMSF) for the protein were assessed for both trajectories produced at pH 5.0 and pH 7.0.

#### 2.4.3 SPR binding assays

Surface Plasmon Resonance (SPR) spectroscopy using a BioRad ProteOn^TM^ XPR360 Protein Interaction Array System was performed to confirm the Bradford method mannosidase binding assays in section 2.4.1. Activation of the ProteOn^TM^ GLH sensor chip surface (Catalogue number 176–5013) was conducted using an EDC/NHS mediated amine coupling method with a flow rate of 30 µl/min for a contact time of 300 s. BtMan2A at a concentration of 250 μg/ml was used for the immobilisation at a flow rate of 30 µl/min for a contact time of 150 s at pH 4.0 using a 10 mM ProteOn acetate buffered solution. The unreacted NHS ester sites on the chip surface were then blocked with 1 M ethanolamine (pH 8.5) with a flow rate of 30 µl/min for a contact time of 300 s (at 25 °C). This method generally resulted in BtMan2A coupled at response levels >2000 response units (RU). Approximately 0.5% (w/v) of LBG dissolved in degassed 50 mM citrate buffer at pH 5.0 was prepared and filtered through a 0.2 µm filter (Minisart RC 15) (to remove insoluble substrate) for SPR studies. The number average degree of polymerization (DP_n_) and number average molecular weight of the filtered/soluble LBG were determined by estimating reducing-ends from the polymer using the bicinchoninic acid (BCA) method as described elsewhere [28], [29]. The mass of the reducing-ends (mg) of the polymer was divided by the quantity of polymer in mg to give the DP of the polymer. The number average molecular weight (*M*_n_) of the polymer was estimated by multiplying the DP_n_ by 160 g/mol (mannose repeating unit) and then adding a fifth of the derived mass to account for galactose substituents on mannose within LBG at a ratio of 1:4 [30].

Six LBG concentrations (0.1–1.0 mg/ml) were injected over the mannosidase and reference surface. The analyte, LBG, was injected at a flow rate of 60 µl/min with a contact time of 90 s and a dissociation time of 300 s. The chip surface was regenerated with 50 mM sodium hydroxide at a flow rate of 100 µl/min with 18 s of contact time. Buffer blanks for double referencing were conducted as separate analyte runs at a flow rate of 60 µl/min with a contact time of 90 s and a dissociation time of 300 s. The binding of LBG in the mobile phase to the chip surface was monitored based on changes in the SPR signal (expressed in resonance units; RU) and was recorded as a function of time (expressed in seconds). The data was zeroed using a reference flow cell in the chip and double referenced with the buffer blank injections from BtMan2A interaction results. All adsorption isotherms were imported to the program BIAevaluation (Version 4.1.1) for evaluation with non-linear data fitting using a 1:1 Langmuir binding model.

### 2.5 Analysis of potential galactomannan binding cavities in CmMan5A

The potential mode of galactomannan binding by CmMan5A was determined by first assessing the enzyme structure for potential cavities that can bind the ligand, followed by its molecular docking on these determined cavities. The tunnel lengths and cavity depths in CmMan5A (PDB id: 1UUQ) were determined using Mole v2.5.17.4.24 [31]. The interacting amino acid residues were selected, and the tunnels were evaluated by adjusting the cavity parameters; bottleneck radius, to 1.25 Å and the cavity parameters; probe radius and the minimum depth, to 3 Å and 5 Å, respectively. Docking of a mannohexaose ligand, representing a portion of the galactomannan polymer, was performed using AutoDock Vina-Carb as described in section 2.4.2.

### 2.6 Thin-layer chromatography analysis of locust bean gum hydrolysis by CcManA

A 400 µl reaction mixture containing 2.5 U of CcManA and 0.5% (w/v) of LBG in citrate buffer (pH 5.0) was incubated at 37 °C for 6 h. After enzymatic hydrolysis, the samples were heat-treated for 5 min at 100 °C and undigested galactomannan was removed through centrifugation at 16,060× *g* for 5 min. Approximately 1, 3 and 6 μl of the samples were applied to Silica Gel 60 F254 HPTLC plates (Merck, Darmstadt, Germany). Linear β-1,4-linked mannooligosaccharides of the degree of polymerization (DP) 1–6, 6^1^-α-D-galactosyl-mannotriose (GalMan_3_), 6^3^,6^4^-α-D-galactosyl-mannopentaose (Gal_2_Man_5_) were used as standards for TLC analysis. The plates were developed twice with 1-butanol: acetic acid: water (2: 1: 1, v/v/v). The sugars developed on the plate were visualized by heating at 110 °C for 10 min after soaking the plates in Molisch's Reagent (0.3% (w/v) α-naphthol dissolved in sulfuric acid: methanol solution (5: 95, v/v)).

### 2.7 Synergy studies

In the enzyme synergy studies, enzyme mixtures were used in various mannanase to mannosidase combinations (CcManA: BtMan2A and CcManA: CmMan5A), based on a protein mass basis. The binary enzyme mixtures had a total protein loading of 18.5 mg/g of galactomannan. The mannanase loading used in the experiments was based on loadings that release at least 0.5 mg/ml (quantifiable concentration using the DNS assay, reaching about 10% substrate hydrolysis) of reducing sugar per hour on LBG hydrolysis. The experiments were carried out in triplicate with 0.5% (w/v) LBG in 50 mM sodium citrate buffer (pH 5.0) in a 400 µl total reaction volume at 37 °C, with mixing at 25 rpm for up to 1 h. The hydrolysis reaction was terminated by boiling for 5 min at 100 °C to inactivate the enzymes. Hydrolysis controls included substrate (containing only the substrate without the enzyme) and enzyme controls (containing only the enzyme without the substrate).

### 2.8 Influence of BtMan2A loading on CcManA mannanase activity

The influence of the protein loading of BtMan2A (18.5–185 mg protein/g biomass) on the CcManA (18.5 mg protein/g biomass) mannanase activity was determined using 0.5% (w/v) LBG galactomannan content in 50 mM sodium citrate buffer (pH 5.0), in a 400 µl total reaction volume at 37 °C, mixing at 25 rpm for up to 1 h. The hydrolysis was terminated by boiling the samples for 5 min at 100 °C to inactivate the enzymes. Hydrolysis controls included substrate (containing only the substrate without the enzyme) and enzyme controls (containing only the enzyme without the substrate).

### 2.9 Prediction of possible BtMan2A to CcManA protein-protein interactions

Using the FASTA sequence of CcManA (UniProt id: Q9LAJ3), the three-dimensional structure of the enzyme was generated to a PDB format using the SWISS-MODEL web server [32], with a mannanase (PDB ID 2C0H) from the blue mussel, *Mytilus edulis*, used as a template. To generate possible protein-protein interaction complexes of BtMan2A and CcManA, the HDOCK program (http://hdock.phys.hust.edu.cn/), that samples the potent binding modes by Fast Fourier transform (FFT)-based global search optimization method and considers the available homolog information from the PDB in generating binding modes, was utilized.

## 3 Results and discussion

### 3.1 Substrate specificity of mannosidases

The enzymes, BtMan2A and CmMan5A, were tested for their specific activities on different substrates at 37 °C using 50 mM sodium citrate buffer at pH 5.0. The *Bacteroides* mannosidase, BtMan2A, exhibited minimal activity towards the doubly substituted substrate 6^3^, 6^4^-α-di-galactosyl-mannopentaose (0.01 U/mg) and towards 6^1^-α-galactosyl-mannotriose (0.015 U/mg) (Table 1). These findings are similar to those reported by Tailford et al. [11]. An earlier report by Ademark et al. [33] also demonstrated that an *Aspergillus niger* mannosidase was active to a lesser extent towards substituted mannooligosaccharides compared to non-substituted ones. Interestingly, BtMan2A was partly active on LBG galactomannan (0.41 U/mg) and released about 1% mannose after an hour of incubation. The ability of BtMan2A to hydrolyse galactomannan substrates is notable, but a more detailed kinetic study is required to fully understand the enzyme's mode of action.

**Table 1:** Specific activities of the mannanolytic enzymes on mannan substrates. Values are represented as means ± SD(n = 3), + low activity; ++ moderate activity; +++ high activity; % is the degree of polymeric substrate hydrolysis; nd = not detected and – = not determined.

Substrate	β-mannosidases (U/mg protein)		β-mannanase (U/mg protein)
	BtMan2A	CmMan5A	CcManA
G2M5	0.01 ± 0.004	0.041 ± 0.003	nd
GM3	0.015 ± 0.001	0.32 ± 0.002	nd
GG	nd	0.70 ± 0.06 (2.3%)	4.63 ± 0.09 (7.1%)
LBG	0.41 ± 0.06 (1.3%)	0.71 ± 0.05 (2%)	9.61 ± 0.19 (11.6%)
*p*NPG	nd	nd	nd
*p*NPM	0.44 ± 0.0	0.19 ± 0.0	nd
M2	+^a^	+^b^	–
M3	+++^a^	++^b^	–
M4	+++^a^	+++^b^	–
M5	++^a^	–	–

1 U represents the release of 1 μmol of product per minute unless otherwise stated.

a – data obtained from[11].

b – data obtained from[15].

Analysis of substrate specificity of CmMan5A (Table 1) indicates that among the considered *p*NP-based, oligomeric and polymeric substrates (guar gum and locust bean gum), this enzyme appeared to prefer polymeric galactomannans as its substrate. Among the galactose-containing oligomers, CmMan5A prefers 6^1^-α-galactosyl-mannotriose (0.32 U/mg) over 6^3^, 6^4^-α-di-galactosyl-mannopentaose (0.041 U/mg). A similar study by Dias et al. [15] showed that hydrolysis of 6^1^-α-galactosyl-mannotriose by CmMan5A released only a single mannose per substrate molecule, and the enzyme displayed no activity against 6^3^, 6^4^-α-di-galactosyl-mannopentaose. This indicates that CmMan5A can accommodate a galactose side chain in the +2 subsite [15]. These findings are similar to those previously reported for the *Myceliophthora thermophile* derived mannosidase, bMann9, by Dotsenko et al. [34].

In this study, the *C. mixtus* mannosidase, CmMan5A, was less active on *p*NPM than the *B. thetaiotaomicron* mannosidase, BtMan2A. These findings were in agreement with those reported by Tailford et al. [11]. CmMan5A was also demonstrated to be more active on the galactose-containing oligomers and galactomannan polysaccharides compared to BtMan2A. For GH5 mannosidases, such as AcMan5, CmMan5A and Op5Man5, it is generally known that for mannooligosaccharides with a DP of 2 to 6 (M2, M3, M4, M5 and M6), the specific activity of the enzymes increases with increasing DP [15], [16], [35]. Our finding for BtMan2A, is similar to reports for other GH2 mannosidases, which reported that these enzymes exhibit higher hydrolytic efficiency on *manno*-oligomers with a DP of 2 compared to oligomers with a higher DP, with approximately 10 times and 15 times higher activity on DP2 oligomers than on those of DP3 and DP4, respectively [12]. As expected, the mannanase, CcManA, showed higher activity on the partially substituted LBG, compared to the highly substituted GG, with significantly higher activity (an order of magnitude of 5–10×) on these polymeric substrates compared to the two mannosidases; CmMan5A and BtMan2A (Table 1).

### 3.2 Mannosidase adsorption study using the Bradford method

The adsorption behaviour of the β-mannosidases, BtMan2A and CmMan5A, on galactomannan (LBG) and their adsorption isotherms trends were determined by the Bradford method at various incubation time points up to an hour at 37◦C and pH 5.0.

**Fig. 1:**
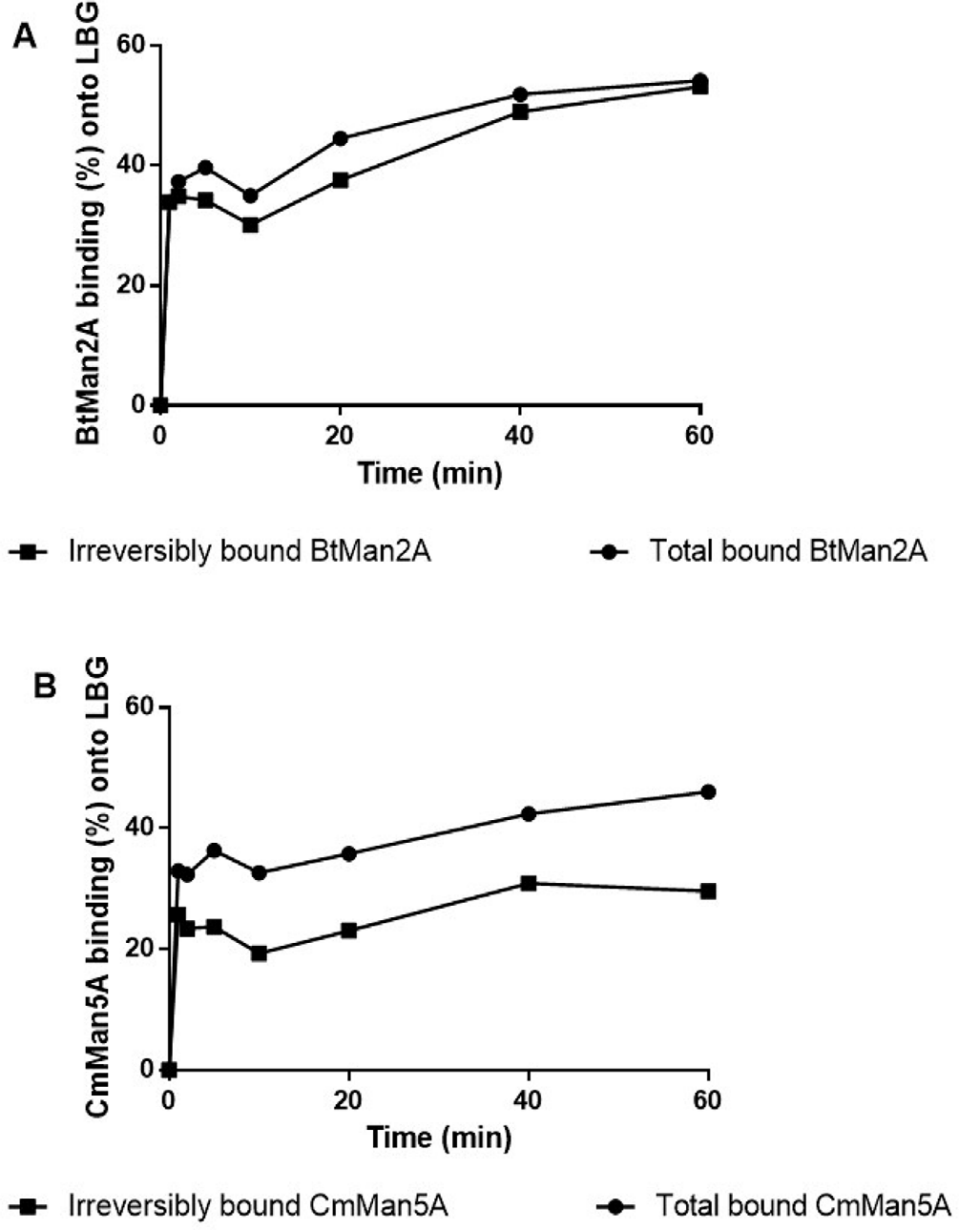
Adsorption isotherms of (**A**) BtMan2A and (**B**) CmMan5A onto LBG, measured by the Bradford method. LBG (5.0 mg/mL) was incubated at 37 °C in 50 mM sodium citrate buffer, pH 5, with each enzyme for up to 1 h.

The enzymes adsorbed immediately to the substrate after being introduced to the reaction, with about 30% of the enzyme mass binding within a minute of incubation for both BtMan2A and CmMan5A (Fig. 1). The rapid adsorption of the enzymes suggested that the mannanolytic enzymes had an affinity for galactomannan (with regards to interaction or binding). During this study, a significant part of the mannanolytic enzyme added initially was bound to galactomannan, with BtMan2A displaying this phenomenon more so than CmMan5A (BtMan2A displayed 52% of total bound protein after 1 h, while about 46% of the initially added CmMan5A protein was bound). With BtMan2A, the percentage of the irreversibly adsorbed enzyme (the enzyme which remained bound even after washing with buffer) was about 51% of the initially added enzyme, with this irreversibly adsorbed amount constituting roughly 98% of the total bound enzyme after 1 h (Fig. 1A). On the other hand, only 29% of the total loaded CmMan5A enzyme was irreversibly adsorbed onto galactomannan after 1 h (Fig. 1B).

### 3.3 Properties of irreversibly bound mannosidases

The enzymatic activity of the irreversibly bound β-mannosidases (BtMan2A and CmMan5A) on galactomannan was evaluated using *p*NPM as substrate. The total irreversibly bound protein on galactomannan after one hour of incubation was compared to the reversibly desorbed protein at the same time point. The reversibly desorbed protein exhibited comparable activity to that exhibited by the original unbound enzyme (kept at the same conditions without mixing with galactomannan) for both BtMan2A and CmMan5A.

**Fig. 2:**
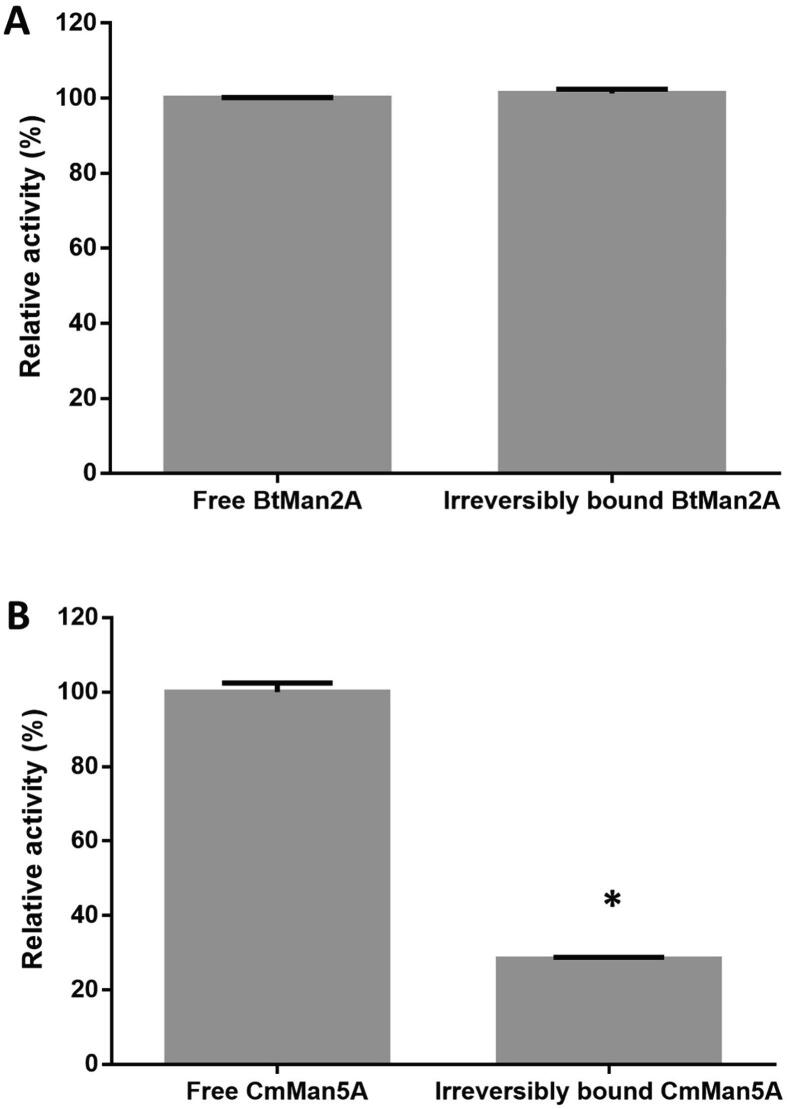
Relative specific enzymatic activity of irreversibly adsorbed (**A**) BtMan2A and (**B**) CmMan5A. LBG (5.0 mg/ml) was incubated at 37 °C in 50 mM sodium citrate buffer (pH 5) with each enzyme. Relative activities of each irreversibly adsorbed enzyme were measured using *p*NPM (2 mM in 50 mM sodium citrate buffer, pH5) as a substrate after 1-hour incubation with LBG. The specific activity of the original enzyme solution kept at 37 °C for 1 h without mixing with LBG was taken as 100%. ANOVA analysis for significant activity difference between the same amount of original free and bound enzyme, key: * (*p* value < 0.05). Values are represented as mean values ± SD (n = 3).

The relative specific activity of irreversibly adsorbed BtMan2A (after an hour of incubation with galactomannan) was the same as that of the original unbound enzyme kept in buffer at 37◦C for an hour (Fig. 2). On the other hand, the relative specific activity of irreversibly adsorbed CmMan5A significantly (p < 0.05) decreased by 70% after an hour, compared to that of the original unbound enzyme kept under the same conditions in the absence of galactomannan (Fig. 2).

From these results, it could be observed that BtMan2A adsorbed onto galactomannan via non-catalytic means, as the enzyme still retained 100% of its mannosidase activity on *p*NPM during its galactomannan bound state. On the other hand, CmMan5A might have bound randomly on the galactomannan substrate – but most likely bound via its catalytic site, as the activity of the bound enzyme on *p*NPM was dramatically decreased. Mole 2.5.17.4.24 showed that CmMan5A has three major cavities, including its catalytic cleft, which could potentially accommodate a galactomannan chain (Supplementary Table A). Docking mannohexaose, to represent a fragment of a galactomannan chain, on these three cavities showed that the catalytic cleft was the only cavity with favourable docking; with a docking score of −8.5 kcal/mol, while the other two identified cavities were inaccessible for docking mannohexaose. This finding supported the postulation that CmMan5A likely binds to LBG via its catalytic cleft, based on the irreversibly bound enzyme activity (Fig. 2).

### 3.4 Effect of pH on BtMan2A binding

**Fig. 3:**
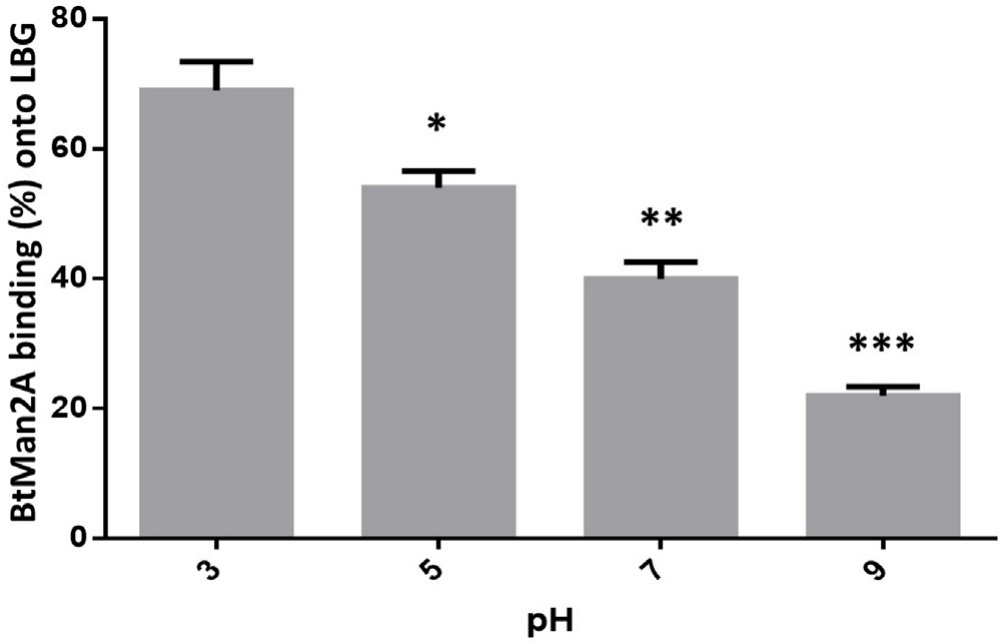
The pH dependence profile of adsorption of the β-mannosidase, BtMan2A, on LBG, measured by the Bradford method. LBG (5.0 mg/mL) was incubated at 37 °C in 50 mM sodium citrate–phosphate buffer (pH 3, 5 and 7) and 50 mM Tris-HCl buffer (pH 9), with BtMan2A up to 1 h. ANOVA analysis for significant differences in binding at different pHs was used, key: * (*p* value < 0.05). Values are represented as mean values ± SD (n = 3).

Similar to what was reported for CbMan2A by Liang et al. [17], BtMan2A galactomannan binding appeared to be pH-dependent, with this binding being more favourable under more acidic pH values (Fig. 3). Kulminskaya et al. [36] also reported that the adsorption of a *Trichoderma reesei* GH2 β-mannosidase on galactomannan was pH-dependent, with its binding also being more favourable under more acidic pH conditions. As mentioned previously, BtMan2A has GH2 conserved galactomannan binding domains, which are rich in histidine residues, which would mediate interactions between galactomannan and the enzymes. As seen with BtMan2A, CbMan2A and *Trichoderma reesei* mannosidase, this effect is most significant at very acidic values of pH (down to pH 3.0). The galactomannan binding ability ceases at neutral pH, when the histidines become non-protonated, which might strongly interfere with the interactions between the enzymes and galactomannan. This is evidenced by the significant reduction of galactomannan binding ability of BtMan2A from 52% to 37% between pH 5 and pH 7 (Fig. 3).

### 3.5 In silico analysis of BtMan2A and galactomannan interaction at varying pH

**Fig. 4:**
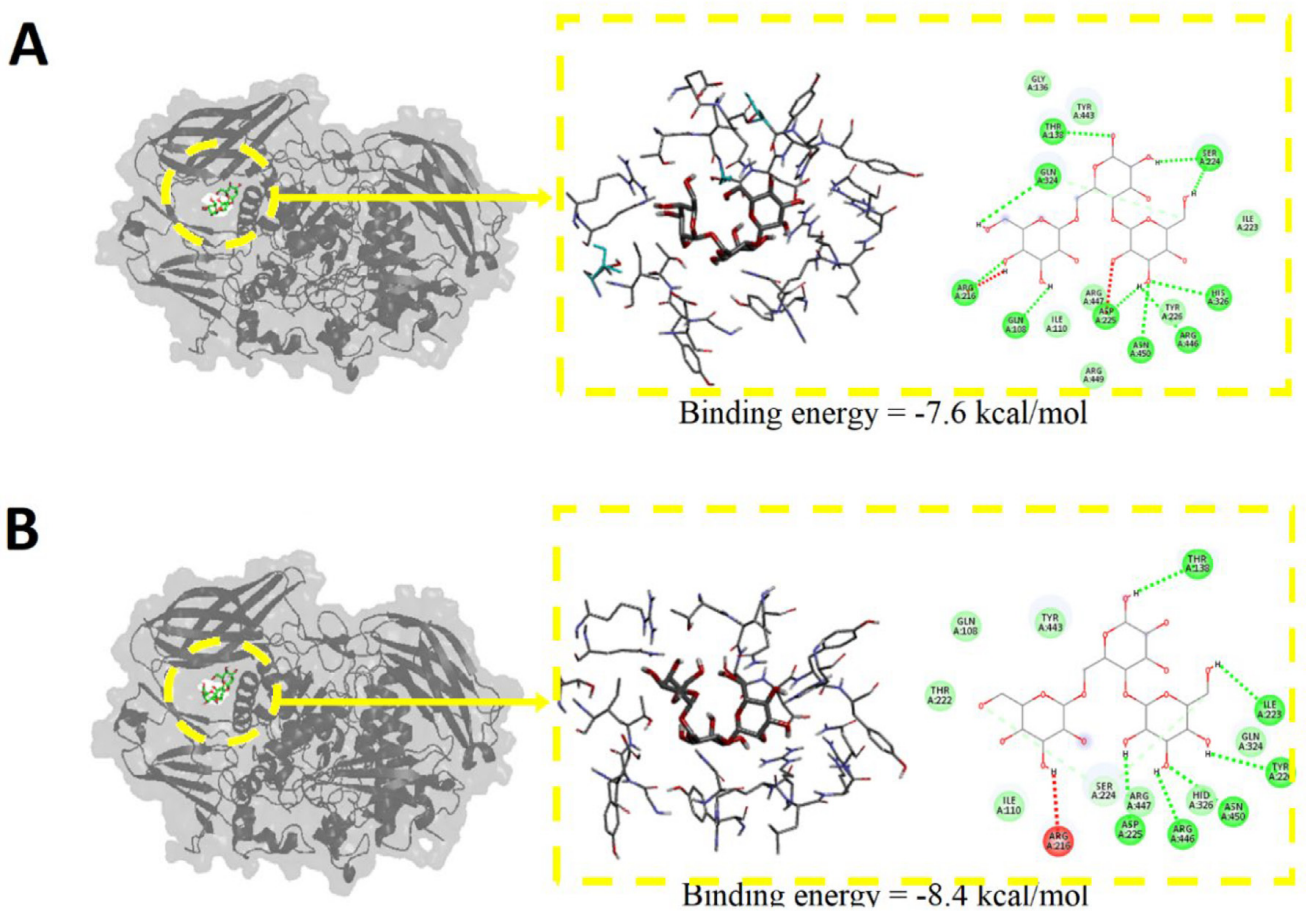
Molecular Docking of BtMan2A at pH 5 and pH 7 using AutoDock Vina-Carb; (**A**) Docking of galactomannan to the putative binding site of BtMan2A at pH 5 showing the interacting residues and (**B**) Docking of galactomannan to the putative binding site of BtMan2A at pH 7 showing the interacting residues, and corresponding binding energies.

To ensure optimal representation of the BtMan2A enzyme during simulation, the Proteins Plus web server was used to protonate the enzyme at the desired pH levels. This resulted in two structures for analysis; one structure protonated at pH 5.0 and the other protonated at neutral pH (pH 7.0). These structures were then subjected to molecular docking using Vina-Carb. The putative binding site was identified by superpositioning the ThMan2A structure with the BtMan2A structure (supplementary Fig. B). The putative binding site was then targeted for docking, and the best identified binding configurations can be observed in Fig. 4. According to the observed binding energies reported by Vina-Carb, the binding of the galactomannan to the BtMan2A structure at pH 5.0 is significantly more favourable as opposed to the BtMan2A structure at pH 7.0. This is evidenced by the BtMan2A binding energy of −7.6 kcal/mol (Fig. 4A) at neutral pH as opposed to the more favourable pH 5.0 binding energy of −8.4 kcal/mol (Fig. 4B). Overall, the galactomannan was bound to the putative binding site of both structures. However, variations in the galactomannan interaction residue network are apparent, where galactomannan binding at pH 7.0 demonstrated more unfavourable amino acid interactions than at pH 5.0, that is 2 versus 1 unfavourable amino acid interaction.

**Fig. 5:**
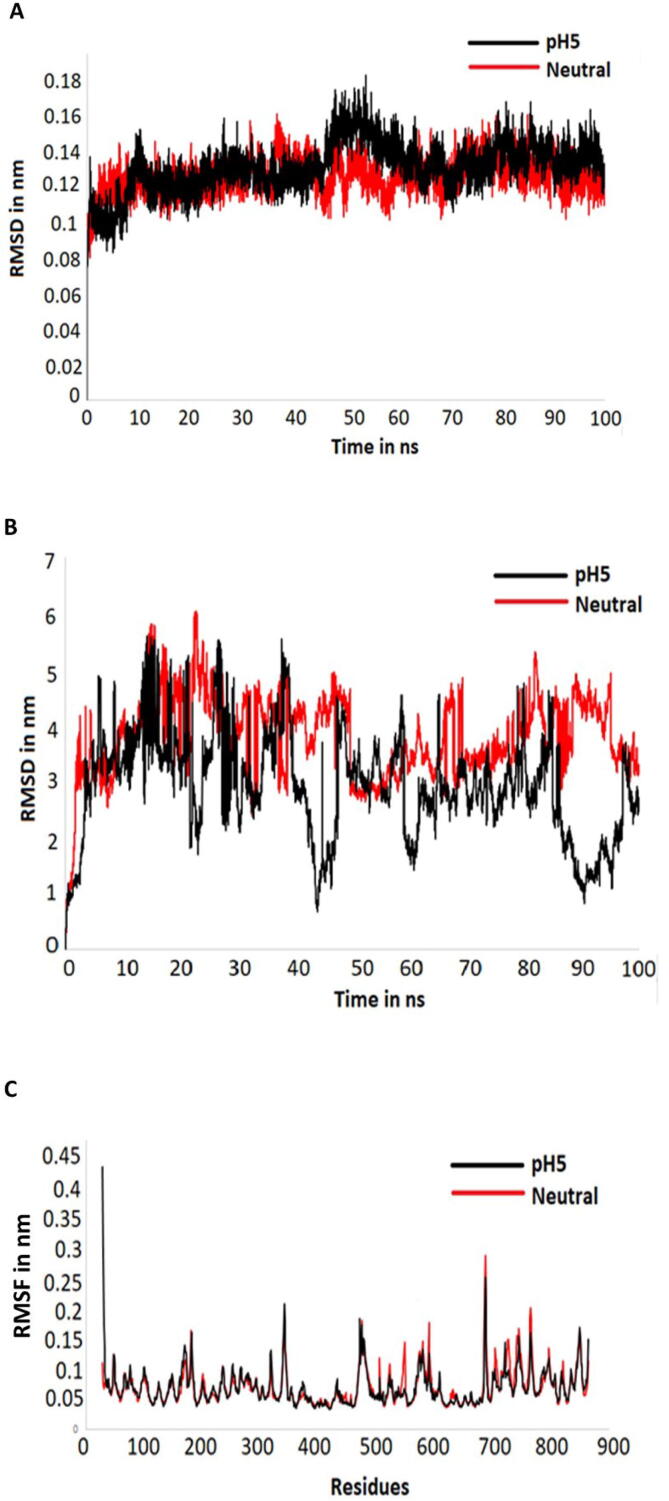
Molecular dynamics simulations of BtMan2A non-catalytic binding domain and galactomannan interaction at pH 5 and pH 7 using GROMACS and the CHARMM 37 force field; (**A**) RMSD analysis of the protein backbone of BtMan2A structures at both pH 5 and pH 7, (**B**) RMSD analysis of galactomannan bound to BtMan2A structures at both pH 5 and pH 7, and (**C**) RMSF backbone analysis of the protein residues of BtMan2A structures at both pH 5 and pH 7.

The stability of BtMan2A to galactomannan interactions at pH 5.0 and pH 7.0 in aqueous environments was assessed using Molecular Dynamics simulations. Each simulation performed had a duration of 100 ns with a 1 fs time step. For each simulation, RMSD of the protein, RMSD of the galactomannan polysaccharide and RMSF for the protein were calculated as shown in Fig. 5. The BtMan26A structure at both pH 5.0 and 7.0 aqueous environments were relatively stable during the simulations, as indicated by the lack of major RMSD fluctuations (Fig. 5A). The behaviour of the galactomannan polysaccharide was monitored throughout the simulations. It was apparent that in both simulations (pH 5.0 and 7.0), there was a high degree of fluctuation in the RMSD of the substrate (Fig. 5B). This fluctuation can be attributed to the number of rotatable bonds found on the galactomannan substrate. These rotatable bonds increase the probability of the molecule rotating during the simulation, resulting in the observed fluctuations. Interestingly, even with the observed instability of galactomannan, the pH 7.0 simulation displayed higher ligand instability as opposed to the pH 5.0 simulation. Low ligand RMSD values observed for pH 5.0 are likely due to the extensive interaction networks that limited the ligand motion, illustrated by the high binding energy. The RMSF calculations showed that the residues (26–221) which interact with the galactomannan substrate appear to be stable throughout the simulation (Fig. 5C). However, the pH 7.0 simulation had a higher degree of fluctuation in the C-terminus residues of BtMan2A, which do not constitute the putative binding site (Fig. 5C). It is unclear how these residue fluctuations may influence the behaviour of the galactomannan binding domain with the substrate.

### 3.6 Evaluation of BtMan2A adsorption isotherms using SPR

**Fig. 6:**
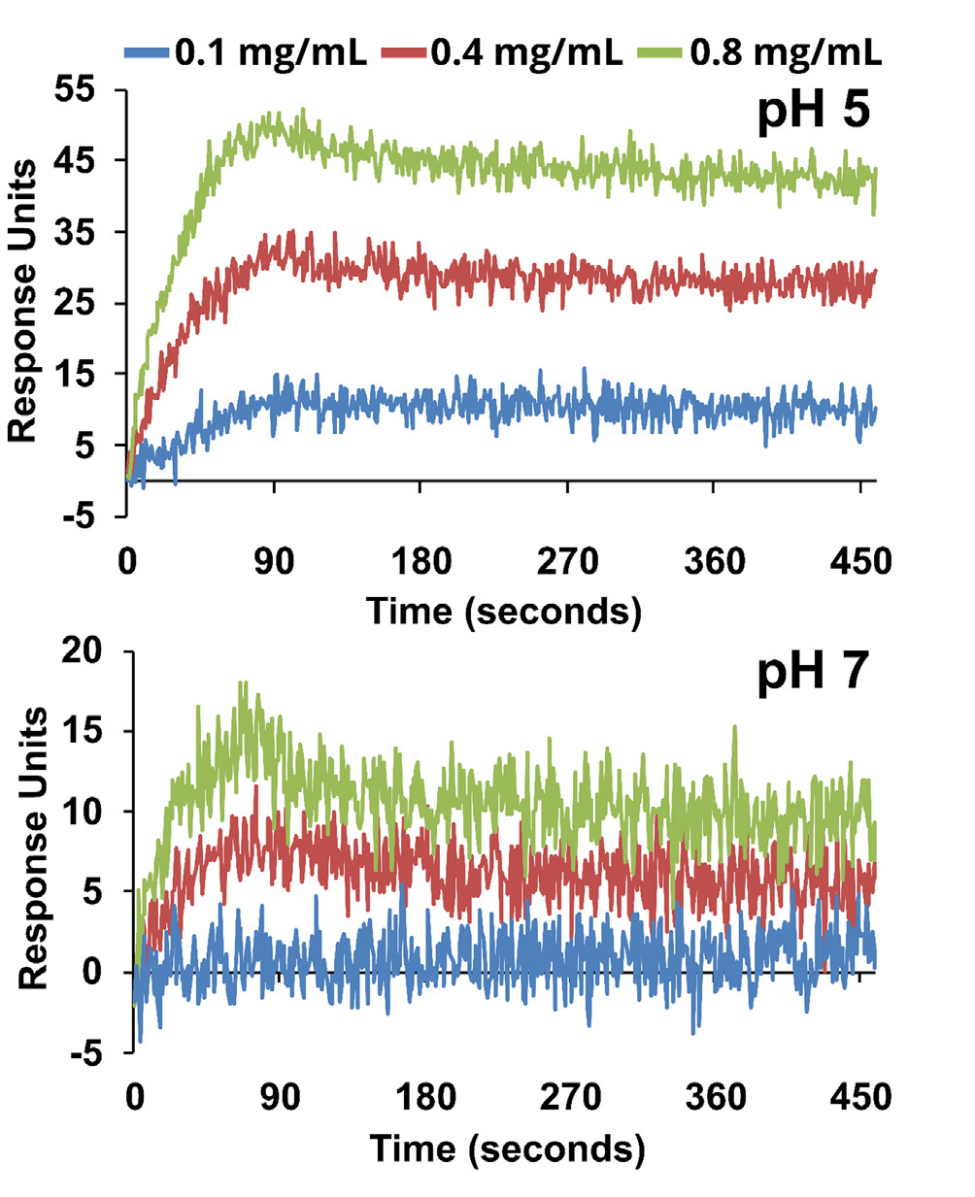
Representative surface plasmon resonance plots of BtMan2A interaction with varied galactomannan concentrations (0.1 to 0.8 mg/ml) at pH 5 and pH 7.

Exploration of the interactions between lignocellulosic components, galactomannan, and mannanolytic enzymes was evaluated by quantification of the proteins remaining in the supernatant once binding of the enzyme protein to the biomass had occurred. The major drawbacks to this approach are (1) interference during protein quantification and (2) the difficulty of distinguishing nonspecific interactions from specific ones [37]. Therefore, we used SPR to qualitatively analyse the interaction dynamics of BtMan2A to LBG (Fig. 6). BtMan2A exists as a dimer in solution [11], with each monomer being approximately 99.5 kDa and the dimer approximately 198 kDa. The soluble fraction of LBG galactomannan used in the binding studies had a DP_n_ of 94 and *M*_n_ of approximately 19.9 kDa. Comparing the binding of galactomannan to immobilised BtMan2A, we showed that there was a substantially reduced binding capacity at pH 7.0 compared to the acidic pH (Fig. 6), supporting the observations from the equilibrium binding experiment shown in Fig. 3. While non-linear data fitting using the 1:1 Langmuir binding model was attempted, the data fit was not ideal.

### 3.7 Thin-layer chromatography analysis of galactomannan hydrolysis by CcManA

Synergism between mannanases and mannosidases appears to be a result of *sequential* action of the enzymes, where mannanases act on the mannan polymers first, leading to the release of short *manno*-oligomers, which are the preferred substrates for the mannosidases. In this section, we, therefore, assessed the types of *manno*-oligomers released by CcManA, which the mannosidases, BtMan2A and CmMan5A, would be exposed to, when used in conjunction with the mannanase, CcManA, during synergy studies for effective galactomannan degradation.

The hydrolysate of LBG galactomannan degraded by the *C. cellulovorans* mannanase, CcManA, was analysed by thin-layer chromatography (Supplementary Fig. C). The mannanase produced various sizes of oligosaccharides, mainly mannotetraose, mannopentaose and some mannotriose, and also *manno*-oligomers with a DP higher than 4, during the hydrolysis of LBG. This indicated that CcManA is a true *endo*-mannanase as no mannose was produced by its action on galactomannan. Another GH5 family mannanase from *Caldicellulosiruptor bescii*, CbMan5A, has been reported to release similar products during the degradation of LBG [38].

### 3.8 Synergy studies

The variable structure and organization of galactomannans require the concerted and synergistic action of various mannanolytic enzymes, such as β-mannanases, β-mannosidases and α-galactosidases for complete degradation [7]. In this study, the two β-mannosidases, BtMan2A and CmMan5A, were assessed for their different galactomannan binding and substrate degradation mechanisms to evaluate how these affect their synergistic interactions with a *C. cellulovorans* β-mannanase, CcManA (GH 5), during galactomannan hydrolysis. The synergistic associations between the enzymes were determined through the quantification of the reducing sugar and mannose released during the degradation of LBG galactomannan. Table 2 summarises the homeosynergy found between the mannanase, CcManA, and the mannosidases (BtMan2A and CmMan5A) during LGB hydrolysis.

**Table 2:** Sugar released (U/mg protein) and the degree of synergy (DS) on LBG by CcManA to BtMan2A and CcManA to CmMan5A.

Enzyme combo	CcManA 100%: BtMan2A 0%	CcManA 75%: BtMan2A 25%	CcManA 50%: BtMan2A 50%	CcManA 25%: BtMan2A 75%	CcManA 0%: BtMan2A 100%
Reducing sugar	9.57 ± 0.04	8.89 ± 0.02	7.12 ± 0.02	3.65 ± 0.03	0.64 ± 0.00
DS _Reducing sugar_	–	0.98	0.94	0.97	–
Mannose	0.07 ± 0.01	0.05 ± 0.00	0.14 ± 0.01	0.35 ± 0.01	0.41 ± 0.01
DS _Mannose_	–	0.89	0.88	1.24	–

Enzyme combo	CcManA 100%: CmMan5A 0%	CcManA 75%: CmMan5A 25%	CcManA 50%: CmMan5A 50%	CcManA 25%: CmMan5A 75%	CcManA 0%: CmMan5A 100%
Reducing sugar	9.36 ± 0.03	11.07 ± 0.03*	9.11 ± 0.03	4.15 ± 0.12	1.29 ± 0.00
DS _Reducing sugar_	–	1.19	1.23	1.14	–
Mannose	0.05 ± 0.02	1.77 ± 0.06*	1.93 ± 0.05*	1.96 ± 0.06*	0.74 ± 0.05
DS _Mannose_	–	2.40	2.62	2.65	–

The specific activity values are presented as means ± S.D (n = 3). DS is the degree of synergy. – not determined. * ANOVA analysis for significant activity difference between combination and the most active single enzyme.

The maximum specific activity (9.57 U/mg protein) produced by the synergistic association of *C. cellulovorans* CcManA and *Bacteroides* BtMan2A was observed when CcManA was at 100% protein dosage during LBG hydrolysis (Table 2). Among the combinations considered, the degree of synergy for reducing sugar release appeared to be around 1, which implies that there was no cooperation between CcManA and BtMan2A in the liberation of reducing sugars. For mannose release, BtMan2A at 100% protein loading displayed the highest specific activity of 0.41 U/mg protein. The highest degree of synergy (1.24) for mannose release was observed at a protein ratio of CcManA 25% to BtMan2A 75% (0.35 U/mg protein) on LBG (Table 2). Table 2 illustrates that the protein ratio CcManA to CmMan5A at a ratio of 75:25% produced significantly (p < 0.05) higher reducing sugar of 11.07 U/mg protein compared to that liberated by CcManA alone on LBG (9.36 U/mg protein). Reducing sugar release appeared to increase with an increase in the ratio of CcManA to CmMan5A (Table 2). All the considered CcManA to *C. mixtus* CmMan5A combinations liberated higher levels of mannose than CcManA alone (0.74 U/mg protein). Mannose release appeared to increase with an increase in the proportion of CmMan5A to CcManA in these binary mixtures during LBG hydrolysis (Table 2).

In general, when inspecting the mannanase to mannosidase (BtMan2A or CmMan5A) combinations, the highest reducing sugar content was found with a mixture of 75% mannanase and 25% mannosidase. Charrier and Rouland [39] reported a different result from this study, namely that the best synergistic effect was found with a mixture of 25% mannanase and 75% mannosidase. We propose that the high content of small fragment products, such as *manno*-oligosaccharides with non-reducing ends, released by the high protein proportion of β-mannanases in the enzyme combination, were the preferred substrates of the β-mannosidases. This may be the reason for the trend observed in synergy between these two main-chain cleaving enzyme classes.

The GH5 mannosidase, CmMan5A, synergistically interacted with mannanase, CcManA, to a greater extent than the GH2 mannosidase, BtMan2A, during galactomannan hydrolysis. This result was in agreement with a previous study that reported the observation of no synergy when a mannanase and GH2 mannosidase combination was used *simultaneously* during mannan hydrolysis [40]. Similar to the observed CmMan5A and CcManA synergism during LBG hydrolysis, a recent study showed Lrman5A, a GH5 mannosidase, and a GH5 β-mannanase synergised during hydrolysis of the same substrate [41]. In the same study, the sequential reaction (β-mannanase → Lrman5A) showed a stronger synergistic effect in the release of mannose than during the simultaneous reaction of the two enzymes. The reason for this may be that small molecule products of the mannanase were more prone to hydrolysis by the mannosidase. In this study, we demonstrated that a GH2 mannosidase, BtMan2A, can bind to galactomannan more effectively than a GH5 mannosidase, CmMan6A, via putative non-catalytic binding sites made up of polar pockets that can recognize and accommodate galactomannans. We previously proposed that this non-catalytic binding ability of GH2 mannosidases to galactomannans competes and interferes with mannanases for their catalytic binding sites on galactomannans, leading to an anti-synergistic behaviour observed between the two enzymes when they are used *simultaneously* [7]. However, in this study, BtMan2A and CcManA demonstrated no synergy (DS ≈1) during galactomannan degradation, demonstrating that the one enzyme did not contribute to the other enzyme's activity on the substrate.

Also, to the best of our knowledge, Liang et al. [17] reported CbMan2A as the first bacterial GH2 β-mannosidase that can bind to galactomannan, yet exhibit strong synergism with CbMan5A in releasing mannose from mannan, with a DS of 1.24. CbMan5A has been reported to mainly release mannose, mannobiose, and other *manno*-oligosaccharides [38], whereas the *C. cellulovorans* mannanase, CcManA, used in this study, mainly releases mannotetraose, mannopentaose and some mannotriose, and also *manno*-oligomers with a DP higher than 6. This difference in hydrolysis products could be the key to the difference between these mannanases in their synergism with GH2 mannosidases, with the mannanase that produces low DP oligomers (CbMan5A) showing synergism with these short DP specific mannosidases of GH family 2. Interestingly, a recent study showed that XacMan2A had a clear preference for longer oligosaccharides [14]; it may be worth investigating how this specific GH2 mannosidase interacts with mannanases during mannan degradation.

### 3.9 Influence of BtMan2A loading on CcManA mannanase activity

**Fig. 7:**
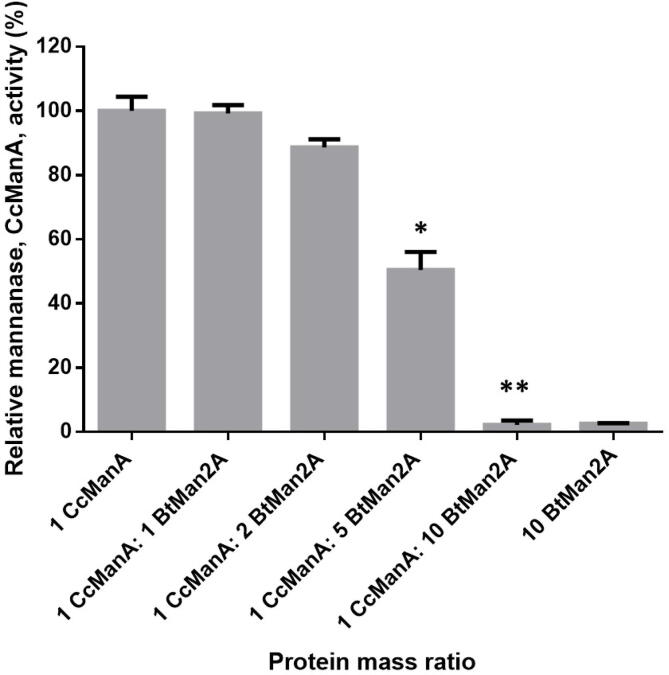
The impact of increased BtMan2A loading (up to 10 parts) on the mannanase activity of 1 part of CcManA during LBG degradation, whereby 1 part of the enzyme is 18.5 mg protein/g of LBG. ANOVA analysis for the decrease in the hydrolysis concerning reducing sugars by the enzyme combinations of CcManA and BtMan2A compared to 1 part of CcManA, key: * (*p* value < 0.05). Values are represented as mean values ± SD. (n = 3).

We also evaluated how saturated loadings of BtMan2A may have an impact on mannanase activity, at a fixed amount (18.5 mg protein/g biomass) of CcManA, during galactomannan degradation (Fig. 7). Our results showed that BtMan2A at loadings <37 mg protein/g biomass did not have a negative influence on the mannanolytic activity of CcManA. At these protein loadings, the possibility of the lack of synergism between the two enzymes (resulting from BtMan2A galactomannan binding ability leading to a blockage of potential hydrolysis sites for CcManA) is avoided. However, increasing the loading of BtMan2A to above 2 parts (>37 mg protein/g biomass) of CcManA exhibited inhibitory effects on the mannanase activity of CcManA during LBG degradation, with 185 mg protein/g biomass load of BtMan2A leading to a complete abolishment of CcManA mannanase activity on LBG. The structural homolog of BtMan2A, the *Trichoderma harzianum* mannosidase, ThMan2A, has been reported to have transglycosylation activity [12]. We suspect BtMan2A at saturated protein loadings may also be displaying this characteristic, hence the decrease in CcManA activity during galactomannan degradation.

The possibility of excess BtMan2A macromolecules interacting with CcManA was evaluated using HDOCK. The most favourable BtMan2A-CcManA interaction complex had a binding affinity and LG-score of -305.79 kcal/mol and 4.117, respectively. The LG-score indicates the reliability of the docking results and was calculated by ProQ program, with protein-protein interactions yielding scores between 3.0 and 5.0 being considered good. This confirmed that there is a likelihood of the two proteins interacting in solution and that the generated interaction complex was good. Assessing the protein-protein complex, it was clear that the interaction complex likey does not compromise the catalytic ability of CcManA, as its catalytic cleft was not involved nor blocked by the interaction between the two proteins (Supplementary Fig. D). furthermore, docking mannohexaose in the active site cleft of CcManA (-8.7 kcal/mol) in the protein-protein complex of BtMan2A and CcManA gave a favourable and similar affinity compared to that observed when docked on free CcManA (-7.5 kcal/mol) (Supplementary Fig. D). Therefore, we concluded that it is unlikely that protein-protein interaction between the two proteins is the cause of the drastic loss in CcManA activity in the presence of saturated BtMan2A concentrations.

Interestingly, as a gut residing bacterium derived enzyme, BtMan2A has been shown to hydrolyse glycans derived from both dietary mannan polysaccharides and host mannose containing glycoproteins as major carbon sources of nutrients for the bacterium, *B. thetaiotaomicron* [42]. In Bacteroides, genes encoding glycan degradation associated proteins are usually co-localized in discrete clusters known as polysaccharide utilization loci (PULs) [43]. According to the Polysaccharide-Utilization Loci DataBase (PULDB) in the CAZy database, BtMan2A is part of PUL 9 that also encodes an *exo*-α-sialidase (GH33), sialic acid-specific 9-*O*-acetylesterase (Carbohydrate Esterase 20), β-galactosidase (GH2), and three β-N-acetylhexosaminidases (GH20) [44], which are likely involved in host mannose containing glycoproteins degradation. Also, the fact that no obvious candidate *endo*-mannanases are found in the *B. thetaiotaomicron* genome, may suggest that the bacterium specializes in scavenging mannose from oligosaccharides produced by *endo*-acting glycoside hydrolases, presumably expressed by other colonic microorganisms through the action of BtMan2A. However, it is unclear as to how BtMan2A galactomannan binding ability could interfere with these colonic bacteria derived *endo*-mannanases during mannan degradation.

## 4 Conclusion

BtMan2A was shown to bind to galactomannan via a non-catalytic binding site which does not affect its activity, while CmMan5A bound randomly with an extreme loss in activity as a result. This particular study has shed light on whether ThMan2A is unique in its ability to bind to galactomannan, showing that this binding capability could be more general among GH2 mannosidases. BtMan2A is the second bacterial mannosidase, after CbMan2A, to be confirmed experimentally to bind to galactomannan. For substrate specificity studies, the data obtained for BtMan2A and CmMan5A was similar to that reported in previous studies, with CmMan5A showing higher hydrolytic efficiency on substrates with a high DP, while BtMan2A showed higher hydrolytic efficiency on substrates with a low DP. The mannanase, CcManA, was shown to predominantly produce oligomers with a DP higher than 4 during galactomannan hydrolysis. CmMan5A could act homeosynergistically with CcManA in the hydrolysis of galactomannan due to CcManA producing CmMan5A preferred substrates as its products (oligomers with a high DP). The lack of synergism between CcManA and BtMan2A during galactomannan degradation was a result of CcManA producing oligomers that were not preferred by BtMan2A, and also as a result of the enzymes competing for substrate binding sites on LBG as previously postulated [7] or the transglycosylating activity of GH2 mannosidases [13].

## Declaration of Competing Interest

The authors declare that they have no known competing financial interests or personal relationships that could have appeared to influence the work reported in this paper.
