# Supplementary material for: Analysis of the galactomannan binding ability of β-mannosidases, BtMan2A and CmMan5A, regarding their activity and synergism with a β-mannanase
Source: Comput Struct Biotechnol J. 2022 Jun 17;20:3140–50. doi: 10.1016/j.csbj.2022.06.038 (PMC9232400; doi:10.1016/j.csbj.2022.06.038)
Supplement: Supplementary data [file mmc1.docx]

**Analysis of the galactomannan binding ability of β-mannosidases, BtMan2A and CmMan5A, regarding their activity and synergism with a β-mannanase**

Samkelo Malgas^1,2,*^, Mariska Thoresen^1^, Vuyani Moses^3^, Earl Prinsloo^4^, J. Susan van Dyk^5^ and Brett I. Pletschke^1^

^1^-Enzyme Science Programme (ESP), Department of Biochemistry and Microbiology, Rhodes University, Makhanda 6140, Eastern Cape, South Africa.

^2^-Department of Biochemistry, Genetics and Microbiology, University of Pretoria, Hatfield 0028, Gauteng, South Africa

^3^-Research Unit in Bioinformatics (RUBi), Department of Biochemistry and Microbiology, Rhodes University, Makhanda 6140, Eastern Cape, South Africa.

^4^- Biotechnology Innovation Centre, Rhodes University, Makhanda 6140, Eastern Cape, South Africa.

^5^-Forest Products Biotechnology, University of British Columbia, 2424 Main Mall, Vancouver, British Columbia, Canada V6T1Z4.

^*^Corresponding author. Tel: +27 12 420 4149

E-mail address: [samkelo.malgas@up.ac.za](mailto:samkelo.malgas@up.ac.za) (S. Malgas)

**Supplementary material**


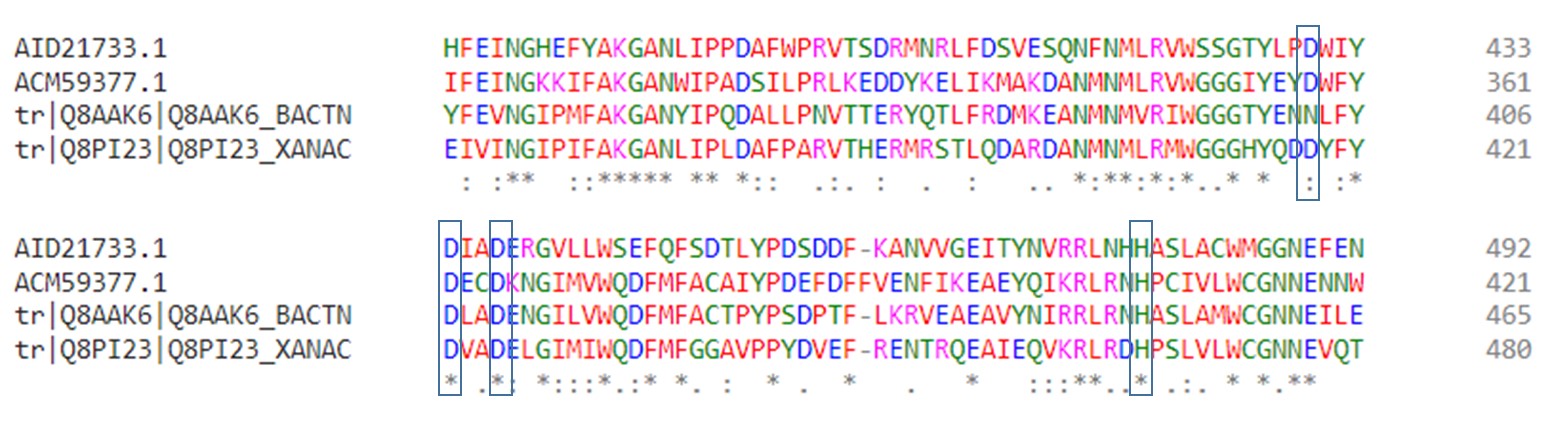


**Figure A**. Amino acid sequence alignment of BtMan2A (Q8AAK6) with those of CbMan2A (ACM593777.1), ThMan2A (AID21733.1) and XacMan2A (Q8PI23). The conserved amino acids in the pocket, potentially involved in galactomannan binding, are indicated by rectangles.


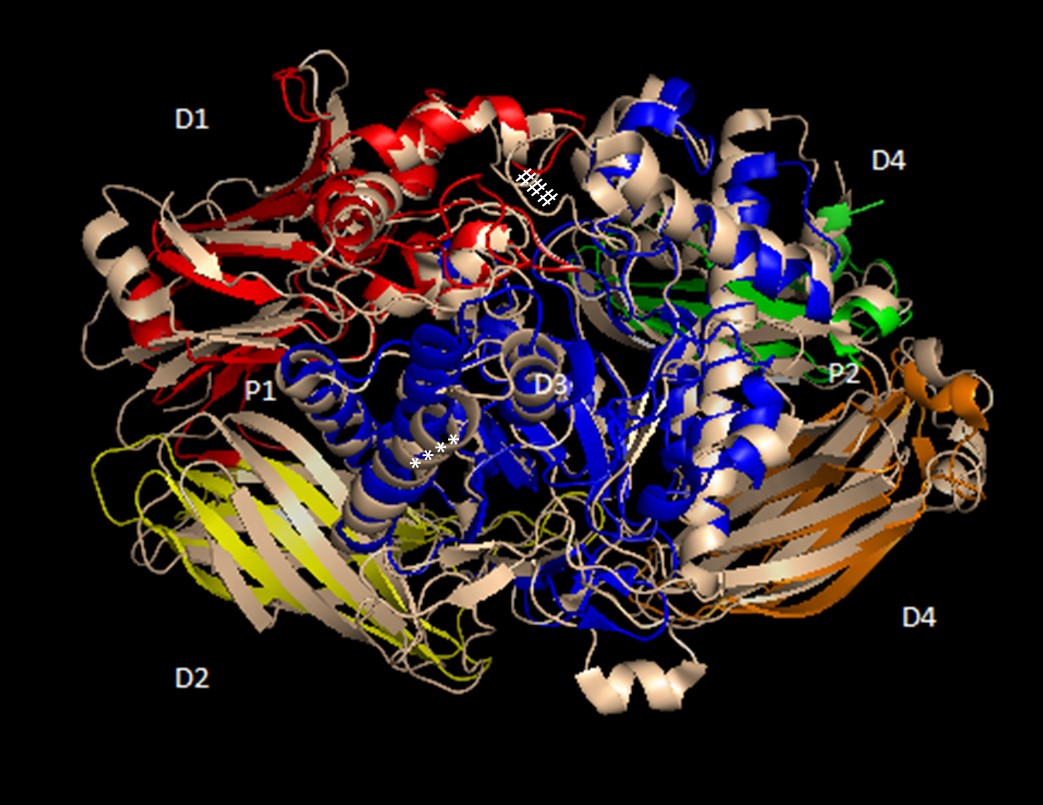


**Figure B.** Superposition of BtMan2A (colour spectrum) and ThMan2A (transparent light grey) using PyMOL. D1-D5 represent the protein domains, while P1 and P2 represent the putative non-catalytic galactomannan binding domains, **** represents a helix movement and ### represents an extended loop.


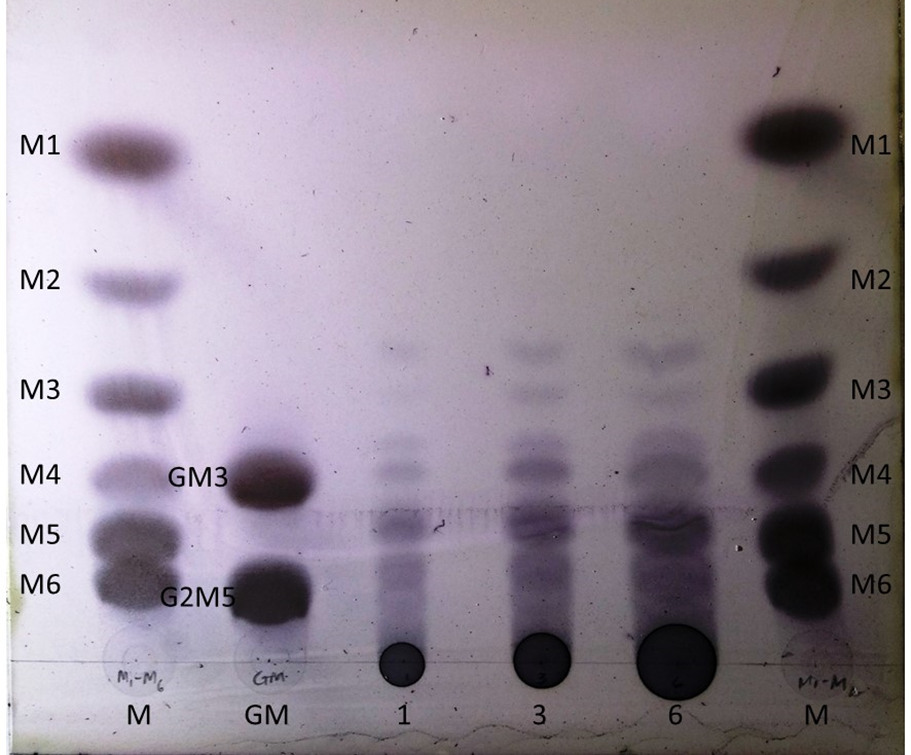


**Figure C.** TLC analysis of the hydrolysis products from LBG. Approximately 5 mg/ml of LBG was incubated with CcManA (2.5 units) and aliquots were loaded at varying volumes (1, 3 and 6 µl), and the hydrolysates were analysed by TLC. A mixture of manno-oligosaccharides (mannobiose to mannohexaose [M2–M6], galactosyl-mannotriose [GM3] and di-galactosyl-mannopentaose [G2M5]) was used as standards.


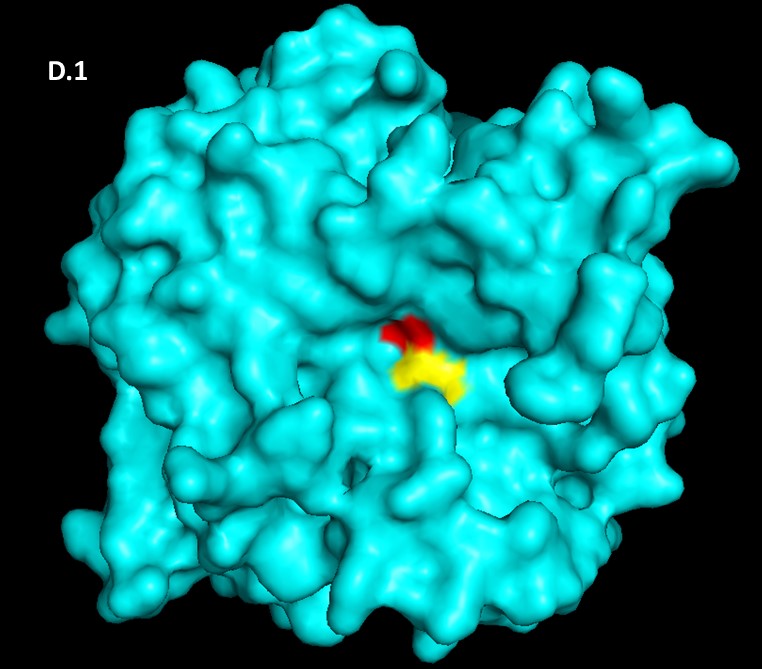

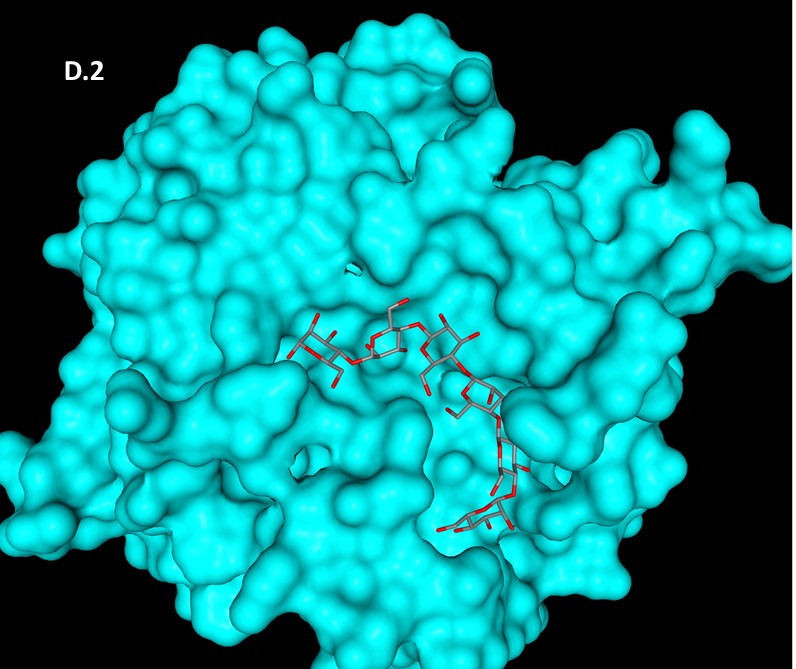


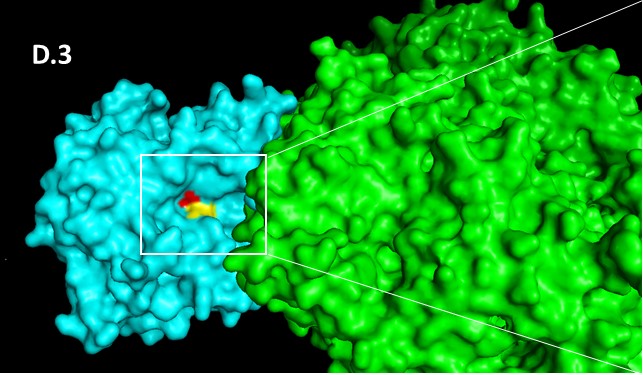

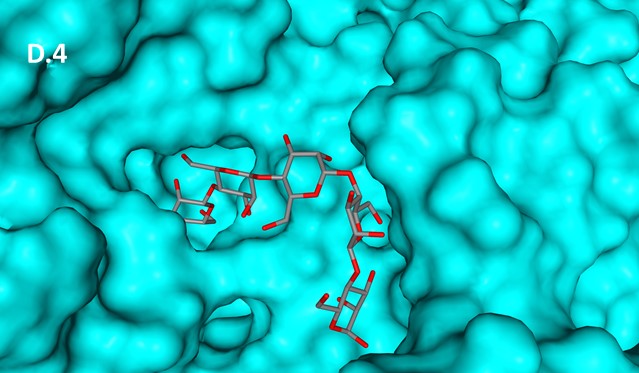


Figure D. (D1) Three-dimensional structure of CcManA (Cyan), with the catalytic residues, E264 and E366, in shown in yellow and red, respectively. (D2) Mannohexaose (colour spectrum) docked in the catalytic cleft of CcManA in CCManA (Cyan). (D3) Complex structure of the mannanase, CcManA (Cyan), against the mannosidase, BtMan2A (Green). The catalytic residues, E264 and E366, in CcManA are shown in yellow and red, respectively. (D4) Mannohexaose (colour spectrum) docked in the catalytic cleft of CcManA in CCManA-to-BtMan2A protein-protein interaction complex (green).

Table A. Identification and molecular docking verification of potential galactomannan binding cavities in CmMan5A

| Cavity | Cavity size (Å^3^) | Cavity residues (inner) | Cavity radius (Å) | Cavity length (Å) | Cavity charge  (inner) | Mannohexaose docking score |
| --- | --- | --- | --- | --- | --- | --- |
| 1 (catalytic cleft) | 276.52 | ASN 45 A, MET 46 A, TRP 47 A, ARG 80 A, TYR 130 A, TRP 137 A, ASN 214 A, GLU 215 A, TRP 285 A, GLU 330 A, PHE 331 A, TRP 376 A, ALA 377 A, GLY 399 A, PRO 401 A, GLN 403 A, TYR 409 A | 2 | 5.39 | -1 | -8.2 |
| 2 | 252.21 | VAL 84 A, GLU 86 A, THR 99 A, GLN 143 A, TYR 144 A, TRP 147 A, TYR 182 A, THR 185 A, LYS 188 A | 2 | 7.99 | 0 | - |
| 3 | 208.11 | ALA 170 A, TYR 173 A, PRO 218 A, GLY 219 A, ILE 230 A, TYR 231 A | 3 | 2.37 | 0 | - |

- no docking possible in the selected cavity
